# Non-*albicans Candida* Species: Immune Response, Evasion Mechanisms, and New Plant-Derived Alternative Therapies

**DOI:** 10.3390/jof9010011

**Published:** 2022-12-21

**Authors:** Manuela Gómez-Gaviria, Uriel Ramírez-Sotelo, Héctor M. Mora-Montes

**Affiliations:** Departamento de Biología, División de Ciencias Naturales y Exactas, Campus Guanajuato, Universidad de Guanajuato, Noria Alta s/n, col. Noria Alta, C.P., Guanajuato 36050, Mexico

**Keywords:** antifungal drugs, host–fungus interaction, antifungal immunity, candidiasis, innate immune sensing

## Abstract

Fungal infections caused by *Candida* species have become a constant threat to public health, especially for immunocompromised patients, who are considered susceptible to this type of opportunistic infections. *Candida albicans* is known as the most common etiological agent of candidiasis; however, other species, such as *Candida tropicalis*, *Candida parapsilosis*, *Nakaseomyces glabrata* (previously known as *Candida glabrata*), *Candida auris*, *Candida guilliermondii*, and *Pichia kudriavzevii* (previously named as *Candida krusei*), have also gained great importance in recent years. The increasing frequency of the isolation of this non-*albicans Candida* species is associated with different factors, such as constant exposure to antifungal drugs, the use of catheters in hospitalized patients, cancer, age, and geographic distribution. The main concerns for the control of these pathogens include their ability to evade the mechanisms of action of different drugs, thus developing resistance to antifungal drugs, and it has also been shown that some of these species also manage to evade the host’s immunity. These biological traits make candidiasis treatment a challenging task. In this review manuscript, a detailed update of the recent literature on the six most relevant non-*albicans Candida* species is provided, focusing on the immune response, evasion mechanisms, and new plant-derived compounds with antifungal properties.

## 1. Introduction

In the last 50 years, we have experienced great advances in healthcare services, which have improved life quality and expectancy. However, this has been accompanied by the increased risk to develop opportunistic infections, such as systemic candidiasis, one of the leading causes of infection-related morbidity and mortality [1]. There are more than 18 different *Candida* species that cause infections in humans, but at least six of these are associated with more than 95% of invasive diseases [2]. Currently, a major part of candidiasis is still owing to *Candida albicans* (63–70%) [3]; however, other *Candida* species, such as *Candida tropicalis*, *Candida parapsilosis*, *Pichia kudriavzevii*, *Nakaseomyces glabrata*, and *Candida auris*, among others, are collectively as important as *C. albicans* in the clinical setting and are known as non-*albicans Candida* species (NAC). Usually, these are found in the environment, skin, or as mucosal colonizers in humans [2,3,4].

**Figure 1:**
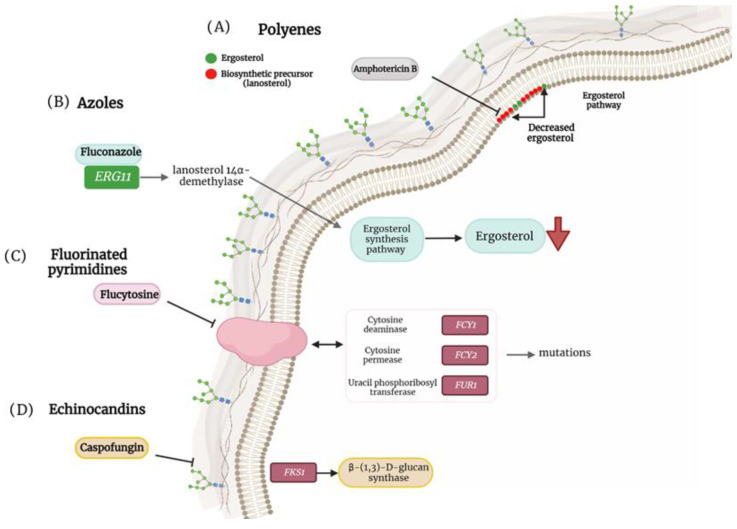
Antifungal resistance mechanisms in non-*albicans Candida* species. (**A**) Mutation in ergosterol biosynthesis that causes a decrease in the ergosterol content in the cell membrane and induces the replacement of biosynthetic precursors such as liquesterol and lanosterol. (**B**) Mutation in *ERG11* that encodes the enzyme lanosterol 14α-demethylase, causing defects in ergosterol synthesis. (**C**) Mutation in cytosine deaminase (*FCY1*), cytosine permease (*FCY2*) and uracil phosphoribosyltransferase (*FUR1*) facilitating resistance to flucytosine. (**D**) Mutation in *FKS1* that generates resistance to caspofungins.

*C. tropicalis* is widely distributed in nature, being a common colonizer of the human skin, oral cavity, and digestive tract. This yeast is an important opportunistic pathogen capable of causing nosocomial infections and it is the second most frequently isolated species after *C. albicans* [5,6]. An important aspect that contributes to invasive candidiasis is drug resistance, and in recent years, this biological trait has increased among the *C. tropicalis* clinical isolates [6,7,8]. The main reason for *C. tropicalis*’ increased drug resistance is due to mutations of the ergosterol synthase encoding gene *ERG11* and overexpression of the transcriptional regulator encoded by *UPC2* (Figure 1) [6,8].

*C. parapsilosis* is often the second or third most frequently isolated *Candida* species in intensive care units (ICUs) since it is capable to form biofilms on central venous catheters and other medically indwelling devices, thus menacing patients who have undergone invasive medical interventions. In addition, *C. parapsilosis*-caused infections are a significant problem among neonates [9,10]. Even though these infections generally result in lower morbidity and mortality rates than those caused by *C. albicans*, diverse clinical isolates of this species have been reported to be less susceptible to echinocandins, and in some regions, resistance to azole treatment has also been noted, which complicates the choice of antifungal drug therapy [10,11,12]. The echinocandin resistance mechanism in *C. parapsilosis* differs from the phenotypic changes seen in other *Candida* species because this fungal species has a natural polymorphism in *FKS1*, which leads to reduced in vitro echinocandin susceptibility (Figure 1) [9,13]. The mechanism for azole resistance is like that described for *C. tropicalis* [6,8].

*N. glabrata* is responsible for nosocomial infections, particularly in adult wards, and is characterized by single biofilm formation, which increases its pathogenesis [2,14,15]. In the United States, approximately one-third of candidiasis cases are caused by *N. glabrata*, causing hematogenous infections [16,17]. Like other NAC, drug-resistant strains of *N. glabrata* are of great concern, as they are being isolated with increasing frequency. For example, in some medical centers, up to 25% of *N. glabrata* isolates are resistant or moderately susceptible to echinocandins [17,18,19]. These data remark on the relevance of *N. glabrata* in the clinical setting. Antifungal drug resistance in this species is related to the over-expression of membrane transporters, point mutations in *ERG11*/*CYP51*, altered sterol import, and genome plasticity, with segmental rearrangements in the M and F chromosomes (Figure 1) [20].

On the other hand, *P kudriavzevii* is an important pathogen in cancer patients, causing both systemic and superficial infections [21]. This organism can cause bronchopneumonia, vasculitis, infections of the tonsils, arthritis, ulcers, and urinary tract, but it is a rare etiologic agent of vaginitis (it has only been isolated in 0.1% of cases) [21,22]. *P. kudriavzevii* has intrinsic resistance to fluconazole and variable resistance to other drugs used in its treatment, including voriconazole, itraconazole, posaconazole, anidulafungin, micafungin, 5-flucytosine, and amphotericin B [23,24,25]. Thus, this changing sensitivity to antifungal drugs makes this organism a potential threat to human health.

As known, *Candida guilliermondii* has become a relevant causative agent of candidiasis in recent years. According to a report by Chen et al., [26] the *Meyerozyma guilliermondii* complex is the second most common *Candida* species isolated from bloodstream infections in a Chinese hospital, and this is the first study that assessed the risk factors, clinical characteristics, and outcomes of candidemia caused by the *M. guilliermondii* complex in cancer patients who have undergone recent surgery. Similarly, this organism has been increasingly isolated in Japan, with a rise in frequency of detection of 14%-24% [27,28,29]. Strains with resistance to azoles, polyenes, and echinocandins have been reported [30]. The most common mechanisms of drug resistance in this species include increased activity of the efflux pump, alteration of the 14 α-demethylase, and point mutations in *FKS1* (Figure 1) [30].

Just over a decade ago, in East Asia, the identification of a new species, *C. auris*, was reported, whose peculiarity was its resistance to fluconazole [31]. Currently, this species has been identified throughout the world, and its relevance relies on the fact that it is difficult to identify in the clinical laboratory, leading to erroneous diagnosis and failure in the treatments, a fact that favors the development of resistance acquisition to multiple drugs [32]. Similar to other *Candida* species, in *C. auris* the molecular mechanisms of resistance to azoles include the overexpression and mutations in the *ERG11* gene (which codes for lanosterol demethylase), and alterations in the sterol synthesis pathway that involves the replacement of ergosterol by other sterols, among others (Figure 1) [32,33]. Mutations in this and other genes, such as *FKS1*, result in elevated minimal inhibitory concentration (MIC) ranges for echinocandins and have been linked to treatment failure (Figure 1) [32,33]. *C. auris* has been classified as a fungal pathogen of concern, which is often associated with nosocomial infections and is considered a threat to human health throughout the world due to its ability to spread efficiently from person to person and cause deadly diseases [34,35,36].

The establishment of *Candida* spp. as a causative agent of tissue and organ damage relies on both the virulence and determinant factors displayed by the pathogen and the host immune response triggered upon tissue invasion [37]. As mentioned, in recent years we have experienced an increment in the frequency of antifungal drug resistance in the different NAC species and *C. albicans*, which poses increased pressure among the scientific community to find new compounds with antifungal properties, with the potential to be used as alternatives to treat candidiasis. Since thorough review papers have been recently published about virulence factors in *Candida* spp. [38,39,40], it results pertinent to conduct an up-to-date literature revision on the interaction of the pathogen–immune cell, the mechanisms to evade host defenses, and the new prophylactic strategies for the treatment of invasive candidiasis caused by NAC species.

## 2. Pathogen–Host Interaction in Different NAC Species

The control of infections caused by the different NAC species, and other fungi, is based on the correct activation of innate and adaptive immune responses [10]. The first step during this interaction is the recognition of the fungal cell wall components. This is a key structure that fulfills very specific functions within the cell, it is responsible for communication with the extracellular environment, and provides strength and protection against host immunity [41,42]. The cell wall has pathogen-associated molecular patterns (PAMPs), which are recognized by pattern recognition receptors (PRRs) located mostly on the cell surface of immune cells [43]. The cell wall PAMPs of the different NAC species are chitin, β-1,3-glucans, β-1, 6-glucans, and *N*-linked and *O*-linked mannans [42,43].

The most studied fungus–host interaction is that of *C. albicans*, and it is thought that this interaction may be similar in NAC species [44]. Recently, several works have reported the immune recognition of different *Candida* species of medical importance [42,45,46]. Immune cells, such as neutrophils and murine phagocytic cells, can distinguish between different *Candida* species. In the case of neutrophils, these show reduced uptake against *P. kudriavzevii*; however, murine phagocytic cells have a greater ability to kill *C. guilliermondii* and *C. krusei* when compared to *C. albicans* [47]. In the same line, *C. tropicalis* is more susceptible to damage by neutrophils than *C. albicans* [48]. Moreover, it has been reported that the interaction of *C. parapsilosis*, *C. tropicalis*, *P. kudriavzevii*, *C. albicans*, *N. glabrata,* and *C. guilliermondii* with human peripheral blood mononuclear cells (PBMCs) is species-specific [42,49].

Analysis of *C. tropicalis*, *C. guilliermondii*, and *P. kudriavzevii* cell walls showed that the composition between the three species is similar [42]; however, this is not necessarily an indicator of a similar interaction profile with components of innate immunity. As proof of this, it was found that *C. guilliermondii* cells have lower levels of β-1,3-glucan, which stimulates low cytokine levels when this polysaccharide is exposed on the surface [42]. Yeast cells of *C. tropicalis*, *C. guilliermondii*, and *P. kudriavzevii* can stimulate higher cytokines levels than *C. albicans* when interacting with human PBMCs and human monocyte-derived macrophages [42]. *C. parapsilosis* shows structural similarities to the *C. albicans* cell wall; however, the arrangement of the components within the wall is different, which has an impact on the ability to activate PBMCs [50].

*C. parapsilosis* is one of the NAC species for which more information has been generated in recent years, including its interaction with the host [10]. For this species, it is known that the Toll-like receptors (TLRs) TLR2, TLR4, and TLR6 are involved in its recognition by gingival epithelial cells, human PBMCs and macrophages [46,50,51,52]. In addition, galectin-3, mannose receptor, and dectin-1 are suggested to be receptors required for responses induced by *C. parapsilosis* [50,53,54]. In recent reports, it has been shown that *C. parapsilosis* stimulates stronger cytokine production than other species, such as *C. albicans*, because of greater exposure of β-1,3-glucan at the cell surface [50]. The disruption of *C. parapsilosis OCH1*, an important gene in the synthesis of the *N*-linked mannan’s outer chain [46], led to changes in the fungal interaction with human PBMCs, stimulating greater levels of IL-1β, in a dectin-1- and TLR4-dependent way [46].

The *N. glabrata* immune recognition has also been studied, and it is known that null mutants with defects in different components of the cell wall, such as β-1,3-glucan or chitin, stimulate a stronger inflammatory response in macrophages [45]. Little is known about the PRRs responsible for *N. glabrata* recognition by macrophages, but dectin-2 is important for host defense against this fungus [55]. A downstream analysis of the PRR signaling pathways determined that the fungus does not induce phosphorylation of the MAP kinases Erk1/2, SAPK/JNK, and p38. However, Syk tyrosine kinase, which signals downstream of C-type lectin receptors (dectin-1 and dectin-2), was activated upon infection of macrophages by *N. glabrata* [45,56]. This organism can survive and replicate in macrophages, which would offer it some advantages, such as immune evasion [57].

A component that plays an important role during the immune recognition of some *Candida* species is phosphomannan [58]. To determine the importance of this wall component in the *C. tropicalis*–host interaction, an *mnn4*∆ null mutant was generated [59]. It was found that cell wall phosphomannans are not required for the stimulation of pro- and anti-inflammatory cytokine production by PBMCs [59]. Assays carried out with human monocyte-derived macrophages showed that the *mnn4*Δ null mutant strain was poorly phagocytosed by these cells [59]. These results are in line with those obtained in *C. albicans*, where the loss of phosphomannan reduced yeast phagocytosis by approximately 50% [60]. However, *C. tropicalis* cells are more phagocytosed than *C. albicans* cells in a dectin-1-dependent mechanism [59].

The mentioned cell–cell interactions play a relevant role in fungal immune recognition; however, there are humoral factors that are responsible for defending the host against NAC species. The complement system is known as a humoral factor of innate immunity against various pathogens. In species such as *N. glabrata*, *C. parapsilosis*, and *C. tropicalis*, the binding of complement proteins has been documented [44,61].

Even though there is considerable progress in understanding *C. auris*’s biological and clinical aspects, its interaction with the host’s immune system is just beginning to be investigated [62]. Previous work has shown that *C. auris* can evade the immune response generated by neutrophils [63]. These immune cells have an important role in the control of fungal infections such as candidiasis. These cells can kill the pathogen through the release of extracellular traps known as NETs [64,65]. It is known that after 4 h of interaction between *C. albicans* and human neutrophils there is an inhibition of cell growth; however, this is not observed when interacting with *C. auris* [65]. Human neutrophils cannot effectively kill *C. auris*, and cell recruitment is poor [63]. This type of immune evasion would have many consequences for those patients who have invasive candidiasis caused by this pathogen [63].

Analysis of cytokine production by human PBMCs established that *C. auris* and *C. albicans* could hardly stimulate TNFα, IL-6, IL-1β, and IL-10 [42]. However, when heat-inactivated cells from the two species were used to stimulate cytokines, higher and similar levels of them were observed [42,50]. In addition, the *C. auris* uptake by human monocyte-derived macrophages is lower when compared to that observed with *C. tropicalis*, *C. guilliermondii*, and *P. kudriavzevii* [42].

*In vivo* and in vitro studies carried out with the *C. auris* clinical isolate BJCA001 demonstrated that once the infection with the fungus is carried out in immunocompetent C57BL/6 female mice, the yeasts can remain in the host and evade the mechanisms of defense [66]. When the fungal load in infected organs was analyzed, increased tissue colonization was observed; however, no morphological changes, such as pseudohyphae or mycelium, were documented [66]. Although there was an increase in colonization, the inflammation and tissue damage suffered by the mice proved to be less severe than the infection caused by *C. albicans* [66]. In line with these observations, interactions with bone-marrow-derived murine macrophages showed a significant increment in the levels of IL-1β, IL-6, TNF-α, CXCL1, and CXCL2 when interacting with *C. albicans* but not when the experiments were performed with *C. auris*, suggesting that the latter is a lesser potent inducer of the MAPK signaling pathway [66]. This reduced proinflammatory response could be related to changes in the β-1,3-glucans masking [66]

Finally, although the knowledge on immunity against different NAC species is an evolving and growing area, it is evident that the immune cell–fungus interaction differs among NAC species.

## 3. Immune Evasion Mechanisms in NAC Species

*Candida* spp. have co-evolved with their host and develop strategies to avoid recognition, thus preventing the onset of inflammatory responses. A well-described evasion mechanism studied in *C. albicans* to avoid recognition by phagocytes is the masking of the immune-stimulatory cell wall β-1,3-glucan layer with mannoproteins [67,68]. The induction of a strong pro-inflammatory response requires the synergistic recognition of β-glucans by dectin-1 and mannans by mannose receptor and TLR4; however, when glucans are buried by mannans the former is hardly recognized by dectin-1, and as a consequence, a poor cytokine profile is stimulated [69,70,71].

Another evasion strategy involves aspartic proteases, which degradate components of the innate immune system, such as salivary lactoferrin, lactoperoxidase, cathepsin D, complement, interleukin-1β, and α_2_-macroglobulin [72]. These proteases are important for cell-wall integrity in *N. glabrata* [71,73]. *N. glabrata* glycosylphosphatidylinositol-linked aspartyl proteases have an important role in activation and survival within macrophages, and are required for virulence, as the expression of these genes is up-regulated upon macrophage internalization [73]. Moreover, for *C. parapsilosis* the presence of genes that encode aspartic proteases involved in the survival within macrophages has been reported [71,74].

Phagocyte escape strategies have been extensively studied in *C. albicans*. Filamentation of phagocytized *C. albicans* cells causes perforation of immune cells, allowing the pathogen to escape [71,75]. In addition to macrophages’ mechanical perforation by *C. albicans* hyphae, pyroptosis, a programmed cell death pathway partially dependent on hyphal morphogenesis, mediates macrophage death following *C. albicans* infection [76]. However, other *Candida* species, such as *N. glabrata*, have different mechanisms when escaping from macrophages. This species survives and replicates within macrophages, unlike *C. albicans*, responding rapidly and robustly to oxidative stress [77]. In addition, it manipulates phagolysosome maturation [57], a feature shared with other NAC species, such as *C. parapsilosis* [74] and *P. kudriavzevii* [78].

Once the host immune response is activated, macrophages, neutrophils, and other phagocytic cells fight fungal pathogens by producing high levels of reactive oxygen species and nitric oxide, resulting in oxidative and nitrosative stresses, respectively. Therefore, the activation of antioxidant responses is the main strategy of the pathogen before internalization by phagocytes. *C. parapsilosis* and *N. glabrata* have the Yap1 protein, which is the ortholog of *C. albicans* Cat1, responsible for the antioxidant systems activation, carbohydrate metabolism, and energy production [79,80]. In its antioxidant function, Yap1 is involved in the induction of conserved genes encoding antioxidant effectors, such as catalase Cta1 [77]. *C. tropicalis* also has a Yap1 protein and fulfills the same function as that described in *N. glabrata* and *C. albicans*, where it regulates the antioxidant pathway of thioredoxin, which protects them from the destruction by neutrophils [80,81,82].

The *C. albicans* Skn7 is another regulator that is required for resistance to hydrogen peroxide [83]. In an analysis by Pais et al. [80], the Skn7 homolog in *C. parapsilosis* was found to be an uncharacterized protein encoded by ORF *CPAR2_304240* and similar to *N. glabrata* Skn7, participates in the response to H_2_O_2_ by inducing the expression of thioredoxins Trx2, Trr1, Tsa1, and catalase Cta1 [84]. In *N. glabrata*, the transcription factors Msn2 and Msn4 are also involved in the regulation of oxidative stress, through the regulation of Cta1, in a concerted action between these proteins with Yap1 and Skn7 [85].

Similarly, regulators of resistance to nitrosative stress have already been studied [86,87], and in the case of *C. albicans*, the best studied is Cta4, a positive zinc finger Zn(2)-Cys(6) regulator that controls the expression of the enzyme nitric acid dioxygenase Yhb1, necessary for detoxification. The *N. glabrata* Yap7 transcription factor acts on Yhb1, constitutively repressing its expression by binding to its promoter [88]. Yap4/6 is another *N. glabrata* transcriptional regulator that participates in resistance to nitrosative stress. In spite of the fact that its ortholog has been identified within the *C. parapsilosis* genome (ORF *CPAR2_11470*), its function remains to be established [80].

The reprogramming of carbohydrate and amino acid metabolism is another aspect that is involved in the persistence and evasion of phagocytes by pathogenic yeasts species [80]. This is because the environment within the phagocytic cells is limited in nutrients, especially nitrogen sources, such as amino acids [89]. It has been reported that Gln3 is a key regulator of nitrogen assimilation in *N. glabrata* since a null mutant strain in *GLN3* showed that the growth rate decreased significantly in all the tested nitrogen sources. In addition, these mutants could not transport ammonium efficiently [89]. In *C. albicans*, the transcription factor Gcn4 plays a key role in the amino acid checkpoint response and is expressed when *C. albicans* is phagocytosed by neutrophils [90,91]. Among other functions, Gcn4 acts on the arginine biosynthetic pathway, which in turn is involved in the production of carbon dioxide and urea, metabolic products that induce filamentation inside macrophages that have phagocytized *C. albicans* as an escape mechanism [92]. In the phylogenetic analysis of Pais et al. [80], it was found that *C. albicans* Gcn4 is closely related to *C. tropicalis* ORF *CTRG_02060*, *C. parapsilosis*, and *N. glabrata GCN4*, suggesting that most likely these NAC species also carry out the evasion mechanism by adapting to amino acid starvation.

Biotin restriction has also been reported to reduce fungal fitness within macrophages, but phagocytosis induces up-regulation of biotin-related *Candida* genes [93]. Specifically, *C. albicans* and *N. glabrata* Vhr1 are important in regulating the short-term responses to the antifungal activities of macrophages and intracellular proliferation. Vht1 becomes relevant for *N. glabrata* replication and early and sustained intracellular growth of *C. albicans* hyphae and hyphal-induced macrophage damage [93].

Neutrophils are known to be predominant cells of the innate immune system that are involved in the control of fungal pathogens such as *Candida* spp. The strategy used by neutrophils to kill fungi is through phagocytosis or the release of NETs. Phagocytosis is effective against unicellular forms of pathogens, such as yeast cells, but for pathogens with larger forms, such as hyphae, NETs are a strategy for their control [94]. However, it has been reported that neutrophils are not able to perform phagocytosis or release NETs against *C. auris*, compared to *C. albicans*, which could suggest that there is an evasion mechanism that has not yet been fully elucidated [63]. Interestingly, another study by Navarro-Arias et al. [42] found that *C. auris* was barely able to stimulate the production of TNFα, IL-6, IL-1β, or IL-10 in human PBMCs, when compared to *C. tropicalis*, *C. guilliermondii*, and *P. kudriavzevii*, which were able to stimulate significantly higher levels of these pro-inflammatory cytokines. This could be a possible immune evasion mechanism that remains to be explored. A further observation about *C. auris* is the ability to form small groups of cells known as aggregates [95], which has been associated with biofilm formation [96]. Both the aggregative and non-aggregative phenotypes induced a minimal inflammatory response in a three-dimensional cutaneous epithelial model. However, when wounding was induced in this model, both phenotypes induced a greater response, but the aggregative phenotype was the most pro-inflammatory [96].

## 4. Development of New Therapeutic Strategies for the Treatment of Invasive Candidiasis Caused by NAC Species

In recent years, fungal infections have been considered a constant threat to the health of immunosuppressed, immunocompetent, and seriously ill patients [97,98]. Infections caused by *Candida* species represent the main cause of opportunistic infections worldwide, which has brought an increase in patient morbidity and mortality [99].

Factors such as the use of broad-spectrum antibiotics and the use of antifungal drugs have increased the frequent isolation of NAC species from clinical samples. Although in recent years there has been a constant development of antifungal drugs that attack the fungal cell wall and the plasma membrane, some of these pathogens have proven to be intrinsically resistant or have acquired resistance to these antifungal drugs, which leads to treatment failure [21,100,101].

Due to this problem, the development of effective and safe therapies is paramount, which can be used alone or in synergy with traditional drugs, to control candidiasis produced by the different *Candida* species [101,102].

### 4.1. Plant Compounds as Antifungal Therapy in Infections Caused by NAC Species

The search for new effective drugs against fungal pathogens has become an increasingly complicated task. Although new medical treatments against mycoses are being developed, specific therapies are limited due to the similarities between human and fungal cells, concerning the structure and biochemical processes [101,103,104]. Thus, the control of *Candida* infections has become a challenge for modern clinicians. Compounds derived from plants are known for the different medicinal properties they offer, including antifungal activities [104]. Unlike commonly used drugs, the manufacture of these promises greater effectiveness and less toxicity in patients [104,105].

Plants have been used throughout the years in different parts of the world for the treatment of diseases. Currently, several reports indicate the efficacy of plant metabolites as possible antifungal agents (see Table 1) [101,104]. It has been shown that the different metabolites help to inhibit fungal growth and alter its virulence [101]. Several plant extracts have been reported in different studies to have activities against *Candida* spp., including *Allium sativum* (garlic), *Cinnamomum verum* (cinnamon), and *Origanum vulgare* (oregano) [106,107,108].

One of the main reasons for the development of anti-*Candida* therapy using plant extracts is that they have unique characteristics, such as their high structural diversity, where primary and secondary metabolites are included [101]. The primary anti-*Candida* metabolites are some peptides and lipids, and the secondary metabolites include alkaloids, terpenes, steroids, phenolic compounds, and other types of organic substances [109,110,111]. Although most of studies have been focused on *C. albicans*, several studies have paid attention to NAC species, which turns out to be promising for this research area (see Table 1).

**Table 1:** Plant-derived compounds with antifungal properties against non-*albicans Candida* species.

Compound	Antifungal Effect	Source	Effect on	References
Primary metabolites Peptides				
*Ca* Thi	Loss of cell viability; increases oxidative stress	*Capsicum annuum*	*C. tropicalis*, *C. parapsilosis*	[112,113]
*Rc* Alb-PepI and *Rc* Alb-PepII	Inhibit biofilm formation; promote biofilm degradation	*Ricinum communis*	*C. tropicalis*, *C. parapsilosis*	[114]
*Tn*-AFLP	Permeabilizes the yeast membrane	*Trapa natans*	*C. tropicalis*	[115]
*Cc* Def3	Inhibits cell growth; permeabilizes the yeast membrane; induces oxidative stress	*Capsicum chinense*	*C. tropicalis*	[116]
*Tes*TI	Decreases ATP levels; induces oxidative stress	*Tecoma stans*	*P. kudriavzevii*	[117]
*Pg*TeL	Decreases ATP levels	*Punica granatum*	*P. kudriavzevii*	[118]
*Pv*D1	Inhibits yeast growth	*Phaseolus vulgaris*	*C. guilliermondii*	[119,120]
Secondary metabolites Phenolic compounds				
Gallic acid	Inhibits the formation of planktonic cells and biofilms	*Buchenavia tomentosa*, *Rosa rugosa*, *Dimocarpus longan*, *Ligusticum mutellina*, *Tamarix gallica*, *Anogeissus latifolia*	*C. tropicalis*, *P. kudriavzevii*, *N. glabrata*, *C. parapsilosis*	[121,122,123]
Caffeic acid	Inhibits the formation of planktonic cells	*Potentilla* sp., *L. mutellina*, *Limonium avei*, *Cirsium* sp., *Olea europea*	*C. parapsilosis*	[124]
Protocatechuic acid	Inhibits the formation of planktonic cells	*R. rugosa*, *L. avei*	*C. tropicalis*	[123]
Cinnamic acid	Inhibits planktonic cell growth	*T. gallica*	*C. parapsilosis*, *N. glabrata*, *C. tropicalis*, *P. kudriavzevii*	[123,125]
Benzoic acid	Inhibits planktonic cell growth	*L. mutellina*, *T. gallica*, *Cirsium* sp.	*C. parapsilosis*, *N. glabrata*, *C. tropicalis*, *P. kudriavzevii*	[125]
Alkaloids and terpenes				
Cinnamaldehyde	Reduces *FKS1* gene expression; reduces ergosterol synthesis	*Cinnamomum verum*	*N. glabrata*	[126]
Thymol	Increases the permeability of the cell membrane	*Thymus vulgaris*, *Origanum vulgare*	*P. kudriavzevii*, *C. tropicalis*	[127]
Geraniol	Inhibits cell growth; regulates the biosynthesis of ergosterol	-	*C. tropicalis*, *N. glabrata*	[33]
Berberine	Modifies the synthesis of ergosterol	*Berberis* sp.	*C. tropicalis*	[128]
Epidihydropinidine	Inhibits cell growth	*Picea abies*	*N. glabrata*	[129]
Yohimbine and vincamine	Inhibits cell growth	*Pausinystalia johimbe*, *Vinca minor*	*C. parapsilosis*	[124]

#### 4.1.1. Potential In Vitro Treatments against NAC Species Developed from Primary Plant Metabolites

The new alternatives for antifungal drugs include antimicrobial peptides (AMPs), which are defined as small molecules that are produced by different organisms, including plants. These molecules have gained special attention due to their potent antimicrobial activity against different organisms, such as viruses, bacteria, and fungi [112,130]. Some of these peptides rapidly kill microorganisms and act in synergism with other AMPs and different common drugs [114]. Unlike other antimicrobial drugs, AMPs have low toxicity to mammalian cells and exert their inhibitory activity at low concentrations [114].

Thionins (Thi) are known as a family of low molecular weight plant AMPs (~5 kDa), which defend plants against different pathogens [112]. Like other AMPs, its activity is based on the interaction with membrane phospholipids, generating membrane instability [131]. Previous reports demonstrated the isolation of Thi from the fruits of the *Capsicum annuum* species, named Ca Thi [113]. Ca Thi showed antimicrobial activity against *C. albicans* and *C. tropicalis*, inducing loss of viability by membrane damage [112]. The analysis of reactive oxygen species production showed that in *C. tropicalis* there was an increase in their production, suggesting that this could be the basis for the growth inhibitory effect [112]. When the synergism between Ca Thi and fluconazole was tested, the combination of the two showed an increase in the inhibitory activity of *C. tropicalis* and *C. parapsilosis*. In *C. parapsilosis*, when Ca Thi and fluconazole were combined, 100% growth inhibition was obtained and for *C. tropicalis,* an inhibition of 96% was achieved. However, when the treatments were used separately, the inhibition was 12%. These data suggest that this synergy could have an important effect in controlling the growth of these two *Candida* spp. [112].

Other peptides used for the possible treatment against *C. parapsilosis* and *C. tropicalis*, identified as Rc Alb-PepI and Rc Alb-PepII, were obtained from *Ricinus communis* seeds [114]. These peptides demonstrated low toxicity to mammalian cells, an important feature that suggests they are effective for their intended use, and showed antifungal activity against *C. parapsilosis* and *C. tropicalis*, inhibiting biofilm formation [114]. In addition, it is thought that both AMPs inhibit adhesion to the extracellular matrix [132,133]. Rc Alb-PepII showed more activity against *C. parapsilosis* (MIC_90_ of 25 µM) and caused damage to the cell membrane, as previously reported for Ca Thi [112,114]. Similar results to those of Rc Alb-PepII and Ca Thi were reported when the Tn-AFP1 peptide obtained from fruits of the *Trapa natans* was used against *C. tropicalis* [115]. From the fruits of the *Capsicum chinense,* an AMP called Cc Def3 was characterized and showed growth inhibition against *C. tropicalis*. The antifungal mechanism was based on promoting the cell membrane permeabilization and induction of oxidative stress [116].

For the treatment of *P. kudriavzevii*, several plant-derived AMPs have been evaluated. Among them is a trypsin inhibitor, which was obtained from the *Tecoma stans* leaves and was named TesTI [117]. Intracellular ATP levels were decreased, and this was associated with mitochondria damage by oxidative stress [117,134]. In addition, TestTI was not cytotoxic when tested in human PBMCs [117]. The PgTeL peptide obtained from the *Punica granatum* sarcotesta also acts as a potential *P. kudriavzevii* antifungal agent [118]. The use of this AMP resulted in a decrease in intracellular ATP and induced lipid peroxidation [118].

*N. glabrata* and *C. guilliermondii* have been studied less in terms of the plant AMP effect. However, different peptides have been evaluated to treat the infection caused by *C. guilliermondii* in vitro [119,120]. Among them, peptides isolated from *C. annuum* seeds have been reported to have antifungal activity against *C. guilliermondii* [113]. These AMP inhibited the growth of this yeast and affected the plasma membrane [135]. In addition, the Pv D1 peptide from *Phaseolus vulgaris* seeds showed antifungal activity against *C. guilliermondii* [119,120].

Interestingly, for *C. auris*, no plant AMP is known to date that can inhibit its growth. However, human AMPs have been described that may be effective against some clinical isolates of *C. auris*, such as histatins, specifically histatin-5 and peptide LL-37 [136].

#### 4.1.2. Potential In Vitro Treatments against NAC Species Developed from Plant Secondary Metabolites

Plant bioactive compounds can act as anti-*Candida* agents due to the cell wall and membrane alterations they cause, especially by reducing the synthesis of ergosterol and polysaccharides [101]. Plants have various secondary metabolites that show this activity, among which are phenolic compounds, alkaloids, terpenes, and essential oils. These have shown promise for the treatment of infections caused by NAC species. Some examples of in vitro treatments from secondary metabolites that have been used to study their possible role as therapeutic agents against NAC species will be described below

##### Phenolic Compounds

Plant families, such as the *Combretaceae* and *Acanthaceae*, have been the most studied as possible therapeutic agents against fungal infections [105]. It has been shown that the leaves, seeds, fruits, and flowers contain the most enriched plant components [105]. Leaves and fruits are the ones that have the highest levels of phenolic compounds; however, their concentration varies depending on the solvent used during the extraction process and its storage [137].

Phenols have shown promising in vitro and in vivo activity against some *Candida* spp. [138,139]. Phenolic acid derivatives, such as gallic, caffeic, cinnamic, benzoic, protocatechuic, and phenylacetic acid, also have antifungal activity [109]. Gallic acid extracted from different plant species, such as *Buchenavia tomentosa*, *Rosa rugosa*, *Dimocarpus longan*, *Ligusticum mutellina*, *Tamarix gallica*, and *Anogeissus latifolia*, has antifungal activity against *C. tropicalis*, *P. kudriavzevii*, *N. glabrata*, and *C. parapsilosis*, with values of MIC ranging between 200 and 12,500 µg/mL, also presenting low cellular cytotoxicity [123]. This acid also can inhibit the formation of planktonic cells and biofilms of some strains of *N. glabrata*, *P. kudriavzevii*, *C. parapsilosis*, and *C. tropicalis* under different MIC values [121,122,140,141]. Concerning caffeic acid obtained from plant species, such as *Potentilla* sp., L. *mutellina*, *Limonium avei*, *Kitaibelia vitifolia*, *Cirsium* sp., and *Olea europea*, it has been reported that it prevents the planktonic cell formation of *C. parapsilosis* with a MIC of 16 µg/mL [124]. The protocatechuic acid obtained from the plants *R. rugosa*, *L. avei*, and *Cirsium* sp is only capable of inhibiting the formation of *C. tropicalis* planktonic cells at a MIC of 400 µg/mL [109]. Cinnamic acid obtained from *T. gallica* has a strong inhibitory potential against the planktonic growth of *C. parapsilosis*, *N. glabrata*, *C. tropicalis*, and *P. kudriavzevii* [109,125]. The last of the phenolic compounds, benzoic acid, obtained from *L. mutellina*, *T. gallica*, and *Cirsium* sp., have antifungal activity against the same species mentioned [125]. These last phenolic compounds were presented as promising candidates to carry out synergies with commonly used antifungal drugs; however, this synergy was only effective against *C. albicans* [125].

The flavonoid (E)-6-(2-carboxyethyl), isolated from *Mimosa caesalpiniifolia*, can inhibit *P. kudriavzevii* growth with an IC_50_ of 44 nM; however, it does not show antifungal properties against *N. glabrata* [142]. In addition, this compound shows synergism with ethyl gallate, reducing the IC levels more than 100-fold [142]. A methanolic extract obtained from *Cynomorium coccineum* showed antifungal activity against *C. guilliermondii* and *P. kudriavzevii*, with a MIC of 0.025 mg/mL [143]. The ethanolic and aqueous extracts obtained from the *Eugenia dysenterica* and *Pouteria ramiflora*, commonly used in Brazil in popular medicine, show excellent activity against *C. tropicalis*, *P. kudriavzevii*, *C. guilliermondii*, and *C. parapsilosis*, with the important characteristic that the MIC values are low [144].

Plant-derived phenols, such as carvacrol, thymol, eugenol, and methyl eugenol, also have antifungal activity against *C. auris*. Carvacrol proved to be the most effective phenol, with no cytotoxic or mutagenic effects in human cells at a MIC of 250 µg/mL. Carvacrol can inhibit *C. auris* adherence to epithelial cells and reduces proteinase production [145].

##### Alkaloids and Terpenes

Other secondary metabolites, such as alkaloids and terpenes, have been studied to establish their potential as antifungal compounds [101]. In some fungi, it has been reported that terpenes can inhibit 3-hydroxy-3-metolglutaryl coenzyme A reductase, as well as cell growth, and trigger apoptosis and cell cycle arrest [102,146]. The terpene cinnamaldehyde obtained from *C. verum* (cinnamon) has fungicidal activity against *N. glabrata* isolates. The terpene caused a reduction in the *FKS1* expression, which is responsible for the biosynthesis of β-1,3-glucan [126] and reduced ergosterol synthesis [126]. The latter was a consequence of the down-regulation of genes involved in ergosterol synthesis, such as *ERG2-4*, *ERG10*, and *ERG11*, and ABC transporters encoding genes such as *CDR1* [101,126].

The anti-biofilm activity of terpenes is behind the efficacy of thymol, geraniol, and carvacrol in the treatment of candidiasis [147]. Thymol (2-isopropyl-5-methyl phenol) is the most abundant constituent of *Thymus vulgaris* and *O. vulgare* [127,148]. This terpene has shown antifungal properties against *P. kudriavzevii* and *C. tropicalis* with a MIC of 39 µg/mL and 78 µg/mL, respectively. In *C. albicans*, thymol binds to plasma membrane ergosterol, increasing ionic permeability, and thus, causing cell death [105,149].

In addition, it was shown that the terpene geraniol is capable of inhibiting the *C. tropicalis* and *N. glabrata* growth with low MIC values, again regulating the ergosterol biosynthesis [33]. Moreover, it also inhibits the plasma membrane proton pump-ATPase of these two fungal species [33]. Geraniol together with fluconazole and amphotericin B show synergy, which causes the growth inhibition of several *Candida* spp. [33].

Some alkaloids have been shown to have terpene-like activity. Berberine, known as an alkaloid derived from plants of the *Berberis* genus, can inhibit the growth of fluconazole-resistant *C. tropicalis* and *C. auris* isolates [128,150]. This compound can modify the ergosterol synthesis and increase the efficiency of the expulsion pumps. These events are more noticeable when there is a synergism between berberine and fluconazole, which would indicate that this alkaloid could be useful in the treatment of *C. tropicalis* [101,128]. Another alkaloid that has been studied for its activity against *N. glabrata* is epidihydropinidine, an alkaloid obtained from the bark of the *Picea abies*. It is not cytotoxic for human cells and the MIC at which it shows the effect is low (5.37 µg/mL) [129]. Alkaloids, such as yohimbine and vincamine, which are obtained from the bark of the *Pausinystalia johimbe* and *Vinca minor*, respectively, have been shown to have antifungal activity against the pathogen *C. parapsilosis* [124]. Thus, terpenes and alkaloids seem to be promising agents against the different NAC species that are frequently found in hospital environments.

##### Essential Oils

Essential oils (EO) obtained from plants have been widely used over time and many properties are currently attributed to them [106]. Thanks to recent studies, it is known that these EOs have interesting antimicrobial properties because of their high content of phenolic derivatives [151,152], and several investigations have focused on the study of the anti-*Candida* activity of different EOs [102,153].

The anti-*Candida* activity of EO derived from cinnamon can inhibit *C. parapsilosis* biofilm formation at a MIC of 250 µg/mL, while fungal growth is inhibited at concentrations of 500 µg/mL [153]. The EO derived from *Cinnamomum tamala* can reduce the biomass of preformed biofilms of *N. glabrata* and *C. tropicalis*, affecting the exopolysaccharide layer of both strains [154].

A wide variety of EOs obtained from different plant species has been evaluated against *N. glabrata*, such as *O. vulgare* (oregano), *Cinnamomum zeylanicum* (cinnamon), *Lippia graveolens* (Mexican oregano), *T. vulgaris* (thyme), *Salvia officinalis* (sage), *Rosmarinus officinalis* (rosemary), *Ocimum basilicum* (basil), and *Zingiber officinale* (ginger) [108]. Through the analysis of these EOs by microdilution techniques, it was found that the EOs of thyme, sage, rosemary, basil, and ginger did not show antifungal activity against this fungal species; however, the EOs of oregano, Mexican oregano, and cinnamon showed antifungal activity [108]. Among the EO that displayed the highest antifungal levels against a group of fluconazole-sensitive *N. glabrata* strains are those from oregano and Mexican oregano, while cinnamon EO showed better antifungal activity against *N. glabrata* isolates that were resistant to fluconazole [108].

EOs derived from different plants were tested against *C. tropicalis*, *P. kudriavzevii*, *N. glabrata*, and *C. parapsilosis* [102]. Among the thirty EOs tested, the oil from *Cupressus sempervirens* (cypress) was shown to have an antifungal effect, acting against all *Candida* spp. evaluated, with variable MICs among species [102]. The EO from *Citrus lemon* showed activity against *C. tropicalis* and *N. glabrata,* with MIC of 250 µg/mL, and the EO from *Litsea cubeba* showed antifungal activity against *P. kudriavzevii* and *N. glabrata*, with MIC of 62.5 and 250 µg/mL, respectively [102].

The EO from *Lippia origanoides* has been evaluated against *C. auris*. Perillyl alcohol and p-cymene showed activity against 90% and 100% of *C. auris* isolates, whereas verbenone, carveol, and trans-β-caryophyllene only showed activity against some strains of this species [155]. Interestingly, strains resistant to the main antifungal drugs from *C. tropicalis*, *C. parapsilosis*, and *C. auris* are the most susceptible to EO derived from *L. origanoides* [155]. In *C. auris*, few studies have evaluated the in vitro activity of EO; however, recent works have evaluated the anti-*C. auris* effect of EO from *Lippia sidoides* [156]. When these EOs are encapsulated in nanostructured lipids, they showed potent activity against this fungal species and were not cytotoxic in in vivo models [156]. The α-cyperone is an EO extracted from *Cyperus rotundus*; it has inhibitory properties on the growth of *C. auris* at concentrations of 150 µg/mL. However, its antifungal mechanism has not been elucidated [157]. Cinnamaldehyde EO extracted from cinnamon bark contains trans-cinnamaldehyde, which is a fungicide that has activity against *C. auris*, and it is likely that the active compound can compromise cell membrane and yeast cell wall integrity [158].

Although many studies are reporting the anti-*Candida* effects of plants, as shown in this work, none of the plant-derived extracts have been tested for use in humans [104]. This could be related to the lack of information on its efficacy, toxicity, or lack of knowledge of its chemical structures. However, these molecules are promising and could help control invasive candidiasis caused by different *Candida* spp.

## 5. Concluding Remarks

Even though research on candidiasis has focused largely on *C. albicans* and the caused infection, other species, such as *C. tropicalis*, *C. parapsilosis*, *N. glabrata*, *P. kudriavzevii*, *C. auris*, and *C. guilliermondii*, have emerged as relevant etiological agents of candidiasis in recent years. The importance of these species relies on their constant isolation in clinical samples, the ability to acquire antifungal drug resistance, and the evasion of the host immune response. The search for new drugs against fungal pathogens has become acomplicated task and specific therapies are increasingly limited, which has challenged modern clinical practice.

Due to these problems, in recent years efforts have been made to develop new effective and safe alternative therapies that can be used alone or in synergy with traditional antifungal drugs. As a result, new bioactive compounds have been identified, coming from primary and secondary metabolites of different plant species. Plants have very interesting characteristics; they naturally contain compounds with antimicrobial properties, and although studies and information are needed to date to use these compounds as a treatment against candidiasis, the findings obtained in vitro open the venue to explore their use in the clinical setting. Unlike commonly used drugs, the use of these bioactive plant compounds promises greater effectiveness and less toxicity in patients.

The development of new therapeutic strategies against the different NAC species will contribute to the control of infections caused by these species and will help reduce the frequency of resistance to common antifungal drugs. Among the challenges to be addressed in this field, we can include the lack of specific chemical structures for some plant-extracted compounds, the development of chemical synthesis to supply the pharmaceutical market, or the sustainable isolation directly from plants.

## Data Availability

Not applicable.
